# Mechanical limits shape the eccentric form of rose prickles

**DOI:** 10.1098/rsif.2025.1300

**Published:** 2026-08-12

**Authors:** Sabrina Gennis, Matteo Pezzulla, Kaare H. Jensen

**Affiliations:** ^1^ Department of Physics, Technical University of Denmark, Kgs. Lyngby, Denmark; ^2^ Department of Mechanical and Production Engineering, Aarhus University, Aarhus, Denmark

**Keywords:** plant biomechanics, elasticity, buckling

## Abstract

Rose prickles are vegetal weapons familiar to most green-thumbed individuals. Their distinctive feature is an oval cross-section, which contrasts with the mostly circular shapes of natural and man-made stingers. However, the reason for their eccentricity remains poorly understood. We explore the hypothesis that the morphology of prickles is physically limited by trade-offs between bending, buckling and cutting. In our experiments, polydimethylsiloxane stingers with varying cross-sectional shapes were used to lacerate a gelatin skin analogue. Circular stingers bend along the direction of travel and create superficial scratches. By contrast, prickles with a moderate aspect ratio have enough mechanical stability to cut (or provide grip). However, when they become too narrow, the blades weaken and buckle. The least unfavourable aspect ratio falls between 2 and 4, not inconsistent with the available biological data (2.7±0.7). This novel mechanical trade-off suggests that cutting mechanics are a potential driver of the rose prickle morphology.

## 1. Introduction

Stingers are among the most widely used weapons in nature [1] and, until the advent of gunpowder, by humans [2]. Most pointed outgrowths have a circular cross-section, presumably because they provide sufficient mechanical stability for puncturing [3]. However, prickles—pointed outgrowths emerging from some plants’ epidermis—are oval, a structurally weaker form. The evolutionary drivers of this apparent inefficiency remain unknown.

**Figure 1.**
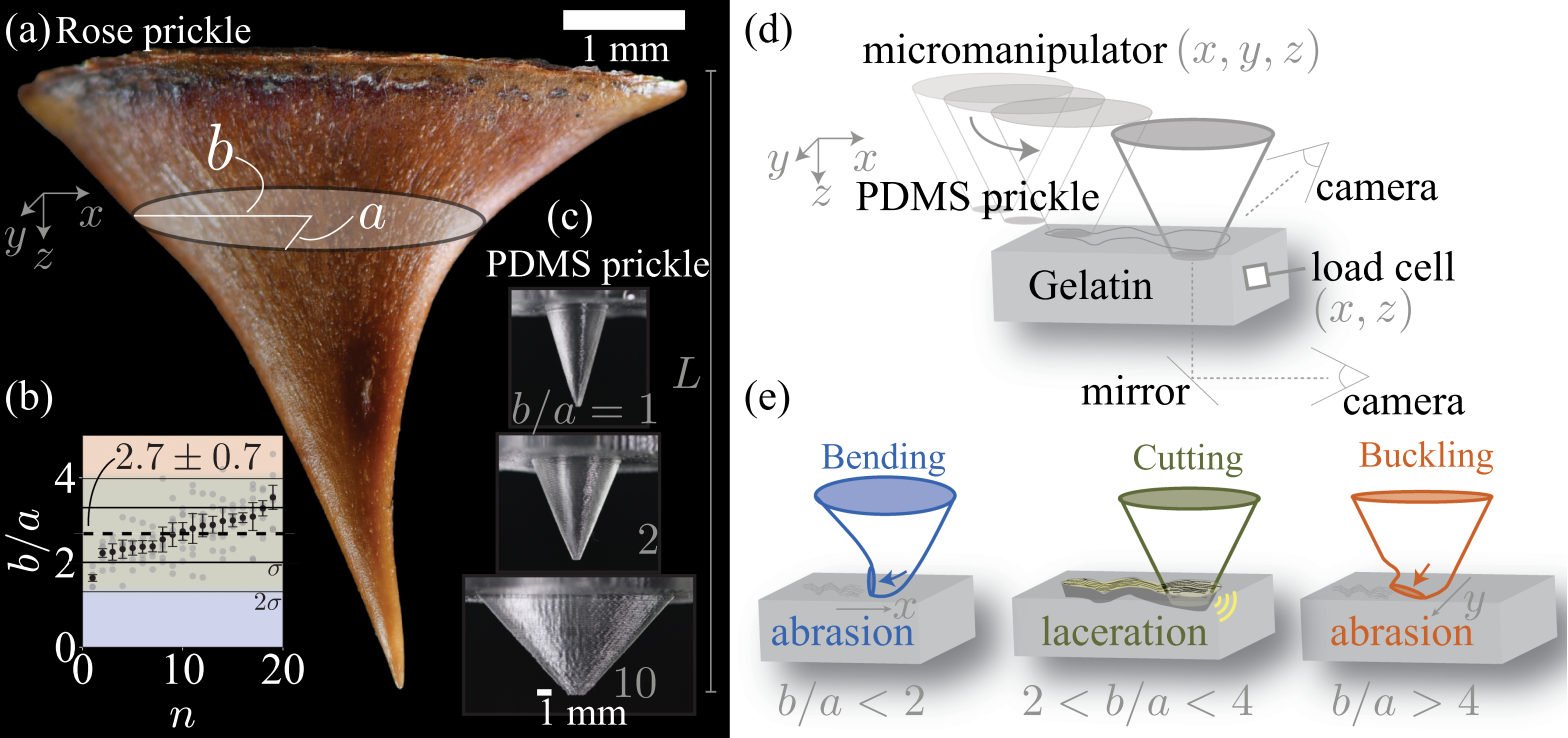
Bending and buckling limit the ability of artificial rose prickles to cut. (a) Prickles serve as a physical defensive weapon employed to deter herbivores. They are well approximated by a tapering elliptical cylinder (aspect ratio b/a, length L). (b) Biological data from 19 rose varieties reveal that a relatively narrow part of morphological space is occupied (mean aspect ratio b/a=2.7, standard deviation 0.7). Sorted by the aspect ratio, the index n identifies the varieties (table S1 in the supplementary material). (c) We use artificial polydimethylsiloxane (PDMS) stingers with varying cross-sectional shapes to explore how prickle shape influences their ability to lacerate a gelatin skin analogue. (d) Artificial stinger motion is controlled using a micromanipulator; cameras and load cells capture qualitative and quantitative data. (e) Biomimetic data (figure 2) suggest that the least unfavourable aspect ratio falls between 2 and 4, where cutting is most frequently observed. Outside this shape range, surface abrasions were the primary observation. In (b), the green-shaded region illustrates that few data points are more than 2 standard deviations (σ=0.7) from the mean. Regions outside these limits are shaded blue and red; see also figures S1 and S2 in the supplementary material.

Members of the Rosaceae (rose) family are perhaps the most widely known examples of prickle-bearing plants (figure 1). Goethe’s 1771 poem *Heidenröslein* [4] vividly describes the potential consequences of attempting to pick a rose: *Ich steche dich, Dass du ewig denkst an mich* (*I will sting, you will never forget*). Roses remain romantic and ornamental plants of choice to this day [5], yet they present an enduring hazard to humans [6].

Prickles serve as a physical defensive weapon employed to deter herbivores [7,8] and thus fill the same functional role as, for example, sponge spicules, cactus spines [9] and hedgehog quills [10]. These examples have an approximately circular cross-section [11,12] and can be represented as a linearly tapering cylinder of base radius a and length L. Members of our team previously proposed that this geometry provides a cost-effective design: circular stinger form can overcome penetrative friction while avoiding buckling [3], and recently Quan *et al.* [12] demonstrated that rotationally symmetric stingers with a parabolic tip are effective for tissue penetration. Analyses of circular cross-section beams have also been used to rationalize the physical limits to tree height [13] and nail shape [14].

Given the abundance of approximately circular plant organs, it is surprising that rose prickles have a tapering elliptical cross-section with an aspect ratio b/a≈2--4 (width 2b, height 2a) [7,15] (figure 1). This design can support a factor of a/b≈1/2--1/4 less force before buckling than a circular stinger of equal length and volume V. That is, it is 50--75% weaker and could, on occasion, fail at penetrating the target tissue. Again, the reasons why rose prickles deviate from the circular morphology remain unclear. Note, however, that while this restricted range is suggestive, it does not in itself imply adaptive selection. Alternative explanations such as sampling bias, developmental constraints or truncated morphospace distributions must also be considered. We will revisit this issue in the Results and Discussion sections.

Prickle orientation and function, however, might hold clues to the physical principles underpinning their distinct shape. The largest diameter (width 2b) of rose stingers is typically aligned with the plant’s orthotropic (vertical) axis. This is not inconsistent with a role in climbing towards the sun, for example on other plants or rocks [7,16]. Moreover, empirical observations by the authors indicate the occurrence of multiple skin lacerations measuring several centimetres in length when managing roses, in contrast to a limited number of puncture wounds. Most such skin tears occur when moving tangentially to the plant stem.^1^ Such interactions arise naturally during handling or when animals brush past vegetation. In mechanical terms, these events correspond to a tangential loading condition in which the prickle tip first engages the surface and subsequently experiences shear as relative motion occurs between the plant and the contacting surface. Shear-driven cutting is known to substantially reduce the force required for tissue rupture compared with purely normal indentation, because it promotes crack initiation and propagation along the surface [18,19]. Consequently, sliding interactions may represent a mechanically demanding scenario that can exert selective pressure on prickle morphology.

A new physical picture is needed if climbing and tearing are primary drivers of rose prickle forms. An elliptical cross-section (b/a>1) mechanically reinforces the structure along the vertical axis, suggesting that narrow prickles (b/a≫1) are expected. However, the observed morphospace is limited to a relatively narrow range b/a=2.7±0.7 (19 varieties; see figure 1 and table S1 in the supplementary material).

In this paper, we explore the hypothesis that the prickle morphospace is bounded by trade-offs between elastic deformation, buckling and the ability to cut. While eccentricity enhances the orthotropic axis of attack, it also weakens the plagiotropic (horizontal) direction and reduces overall buckling resistance.

The paper is organized as follows. We begin by introducing an experimental setup to quantify deformation and cutting dynamics. Varying the eccentricity, we show that at a moderate aspect ratio prickles (b/a≥2) have the mechanical stability to create lacerations and provide grip. However, as it narrows (b/a>4), the blade becomes so weak that it buckles along the orthotropic axis. Data are supported by computer simulations and linear elasticity theory. Our results suggest a rationale for the biological data and provide a new quantitative measure of prickle fitness.

## 2. Methods

We combined biological data with a biomimetic approach, theory and computer simulations to explore the performance of straight elliptical tapering stingers as a function of their geometry. This isolates the effects of shape from, for example, curvature and material properties (Discussion). Detailed experimental and computational protocols are provided in the supplementary material (see also [20–25]). Briefly, rose prickle morphology was quantified for 19 varieties in the Rosaceae family (§S1 and table S1 in the supplementary material). Artificial prickles were produced by casting a silicone polymer (polydimethylsiloxane, PDMS) in a three-dimensional (3D)-printed negative mould. Samples with eccentricities in the range b/a=1--10 were fabricated (table S2 in the supplementary material). (The range b/a=0.1--1 was explored by rotating the synthetic prickles by π/2.) The prickle volume V, length L and tip area $A_{t}$ were kept constant in all experiments. Stingers were mounted on a micromanipulator [26], allowing us to recreate distinct biomechanical scenarios. Herbivory defence was implemented as a two-stage motion mimicking, for example, a human hand grabbing and pulling the plant stem: initially, the prickle approached the substrate at a $\pi /8\approx 23^{\circ }$ angle, until the tip reached a depth of d=1.2 mm. Subsequently, a purely tangential motion (length 5 mm) was executed, resulting in a total tangential displacement $\ell_{0}=7.4$ mm. A skin tissue analogue (gelatin) was used as the target substrate, and glass was used as a rigid control surface. Cuts or abrasions were observed depending on the prickle aspect ratio b/a. The laceration length $\ell_{c}$ (if present) was recorded. The two types of injury were clearly distinguishable by their optical signature: lacerations as deep cuts and abrasions as surface scratches [27]. Force–displacement curves were acquired using load cells, while a camera captured the motion.

Both gelatin and PDMS exhibit viscoelastic behaviour, and the influence of loading rate can therefore be assessed using the Deborah number $De=t_{r}/t_{p}$, where $t_{r}$ is the characteristic relaxation time of the material and $t_{p}∼1$ s is the timescale of the experimental deformation process. The Deborah number quantifies whether a viscoelastic material behaves more like a solid or a fluid during a given process. Gelatin hydrogels of comparable concentration typically exhibit relaxation times of order $t_{r}∼10^{2}$ s, while cured PDMS elastomers behave essentially as elastic solids over laboratory timescales [28,29]. The resulting Deborah number is therefore expected to be larger than unity, indicating that both materials respond primarily elastically during the cutting process. Under these conditions, the geometric trade-offs between bending resistance and buckling derived in this work provide an appropriate leading-order description of the mechanics.

Computer simulations of bending and buckling of tapered elastic beams were carried out in COMSOL Multiphysics (Version 6.2, COMSOL AB, Stockholm, Sweden). Specifically, bending simulations were performed within the Solid Mechanics interface, with a linear elastic material and geometric nonlinearities. The tapered beam was clamped at the top surface, while a displacement field was imposed on the entire bottom cross-section. The Cartesian components of the displacement field in our reference frame (see figure 1) are denoted as (u,v,w). For each value of the displacement field, the reaction force at the clamp was extracted. Buckling simulations were also carried out within the Solid Mechanics interface, but in a two-dimensional, plane strain simplified framework where the indented plane was modelled as a rigid material. A vertical displacement field was imposed at the top surface, while contact was modelled via a penalty method with Coulomb friction. The reaction force at the top surface was extracted for every value of the imposed displacement. Additional details are provided in the supplementary material.

## 3. Results

Prickles are integral to rose plant defence, but their distinct oval cross-section remains poorly understood (figure 1a,b). Here, we consider possible reasons why the elliptical cross-section could be a trait that increases the survival chances of the organism. Specifically, we explore the ability of circular and oval silicone polymer stingers to cut into a gelatinous skin analogue. When juxtaposed with the stingers’ bending and buckling characteristics, as well as computer simulations and scaling laws, we can provide an interpretation of physical trade-offs associated with the prickle shape.

### 3.1. Rose prickle morphology

Our starting point is the morphology of pointed outgrowths emerging from the epidermis of rose plants. Each prickle is unique and may vary in shape and size depending on, for example, its age, position on the plant and on environmental conditions. Anatomical studies have documented such variation in prickle morphology and mechanical properties along rose stems and across developmental stages [16]. However, a general feature appears to be that the width-to-height ratio spans a relatively narrow range b/a=2.7±0.7 (mean ± standard deviation for 113 samples from 19 rose varieties). We cannot rule out that this particular range of b/a could reflect sampling bias, a truncated underlying distribution, or developmental constraints on epidermal outgrowths. In the present study, prickles were sampled across multiple varieties and locations without explicit selection for particular shapes; however, the dataset remains limited and does not constitute a statistical test against a null model [30]. Nevertheless, the following observations render this relatively unlikely: all data (except for two outliers) fall within 2 standard deviations of the mean, and no prickle has an aspect ratio below 1.4 or above 4.6. Moreover, members of the Rosaceae family are fully capable of producing tissues with eccentricities below and above this limited range. For instance, stems and branches have an approximately circular cross-section (equivalently b/a≈1), while leaves approximate a plate-like geometry (equivalently b/a≈100 [31]). It is therefore not impossible that the observed prickle aspect ratio is a consequence of an evolutionary adaptation. We speculate that it conveys mechanical stability to the prickle, enabling it to create lacerations and provide grip. This facilitates defence and climbing, which could increase the survival chances of the organism.

### 3.2. Artificial prickles: cutting mechanics

Having established the characteristics of natural prickles, we now proceed to describe the results of mechanical experiments on artificial prickles. This approach allows us to gauge the relative performance of geometries *not* observed in nature, and hence shed light on processes that could, potentially, incentivize nature to avoid certain parameter ranges. The experiments trace the consequences of polymeric stingers in contact with a gelatin surface during tangential motion.

**Figure 2.**
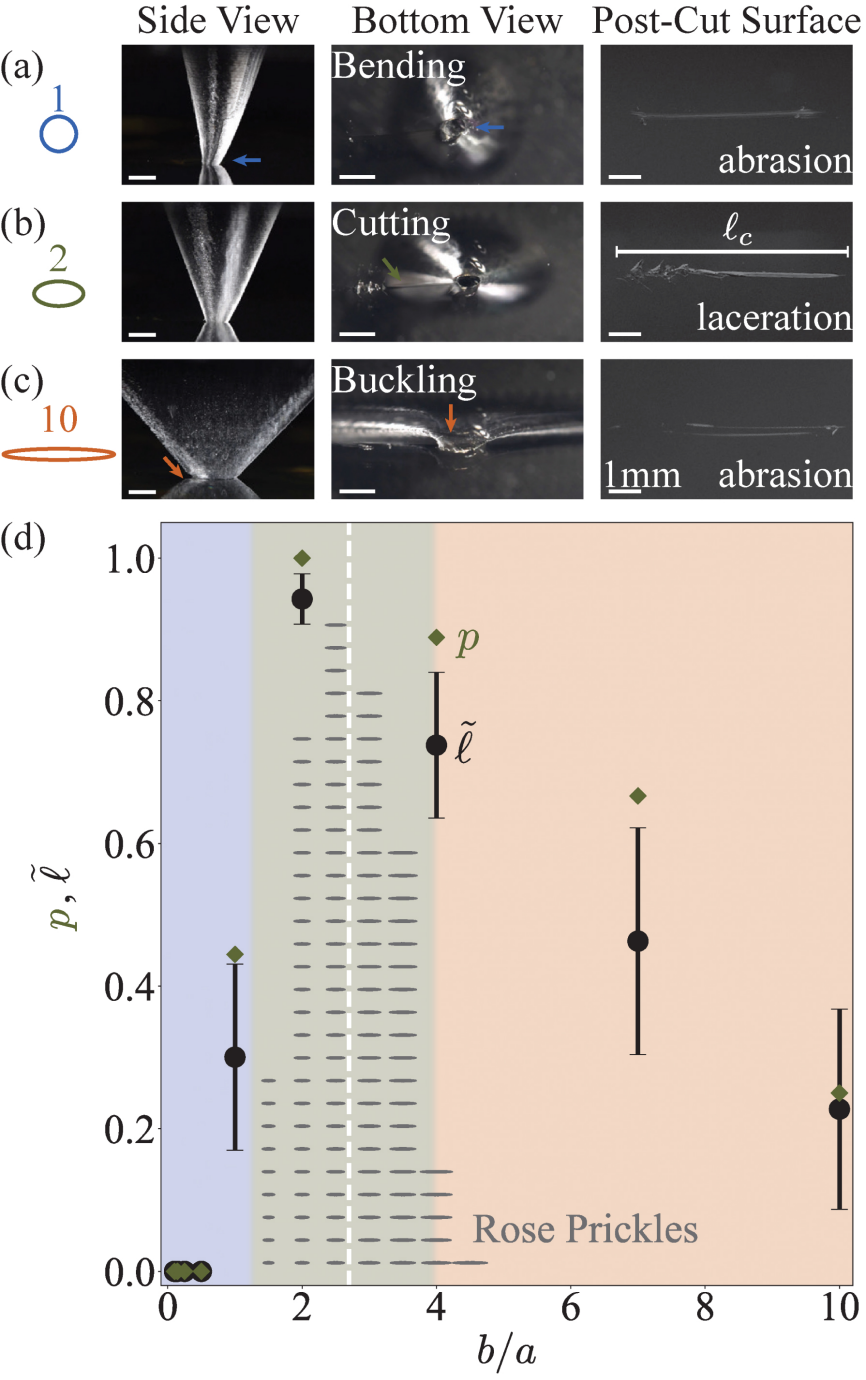
Rose prickle morphology is not mechanically inefficient. (a–c) Still images from the biomimetic herbivory defence process capture the PDMS prickles cutting into a gelatin slab (video 1 in the supplementary material). Circular stingers (b/a=1) bend along the cut direction and leave gentle abrasions on the surface. By contrast, moderately eccentric prickles (b/a=2) are able to resist bending and can leave deep lacerations. Finally, slender stingers (e.g. b/a=10) buckle transversely, leaving the surface largely intact. (d) The cutting probability p (green diamonds) and effective cut length $\tilde{\ell }$ (black circles) are plotted as a function of the prickle aspect ratio b/a. The highest proportion of successful (and/or relatively long) lacerations occurs for aspect ratios in the range b/a=2–4. Data from rose prickle morphology (grey ellipses; table S1 in the supplementary material) cover the interval from b/a=1.4 to 4.7. The dashed vertical line indicates the mean b/a=2.7, and the background is shaded using the colour codes introduced in figure 1b.

We first consider circular prickles b/a=1 (figure 2a). After touching down, contact forces cause the stinger to bend along the x-axis of motion, sliding along the surface. This typically leaves behind a gentle abrasion. By contrast, for moderately elliptical forms (b/a=2), bending is suppressed, and deep lacerations are most frequently observed (figure 2b). This represents a successful cutting or laceration event. Finally, for strongly eccentric geometries (b/a=10), the stinger tip buckles along the y-axis normal to travel (figure 2c). Most often, this results in surface abrasion. Finally, attempts to cut along the weakest axis (b/a<1) never resulted in lacerations.

The outcome of each artificial herbivory defence experiment depends on the detailed conditions, and slight variations in alignment, initial conditions, surface texture, etc., can cause differences in the outcome. To account for this, we repeated each scenario at least six times (§S5 and table S3 in the supplementary material). From these data, we constructed two quantitative metrics. First, the cutting probability p, representing the fraction of experiments that resulted in deep lacerations. Second, the effective cut length $\tilde{\ell }=\ell_{c}/\ell_{0}$, where, as previously mentioned, $\ell_{c}$ is the length of the cut and $\ell_{0}$ is the range of travel along the x-axis.

The cut probability p and normalized laceration length $\tilde{\ell }$ capture the complex dynamics at play when plotted as a function of the stinger form factor b/a (figure 2). This graphical representation reveals a distinct optimum in the prickle performance in the range b/a≈2--4. Cuts are physically unlikely outside this range because the stinger will either bend along the direction of motion (b/a<2) or buckle in the normal direction (b/a>4). Interestingly, a histogram of biological prickle data closely follows this trend, and the mean natural eccentricity b/a=2.7 is relatively close to the aspect ratio that maximizes the cut probability p and cut length $\tilde{\ell }$ in the synthetic experiments.

### 3.3. Artificial prickles: deformation and buckling

To complete the physical picture that may limit rose prickle morphology to a certain aspect ratio b/a range, we end this section by briefly discussing the deformation and buckling of tapering elliptical stingers. The deformation of tapering beams is a topic that has been widely studied previously, and in isolation, the mechanics of the geometries at hand are not novel (e.g. [32–34]). To unpack the detailed structural changes in our biomimetic stingers, however, we carried out mechanical testing. These tests are centred on the bending and buckling and allow us to distil processes that might limit rose prickles’ stability.

**Figure 3.**
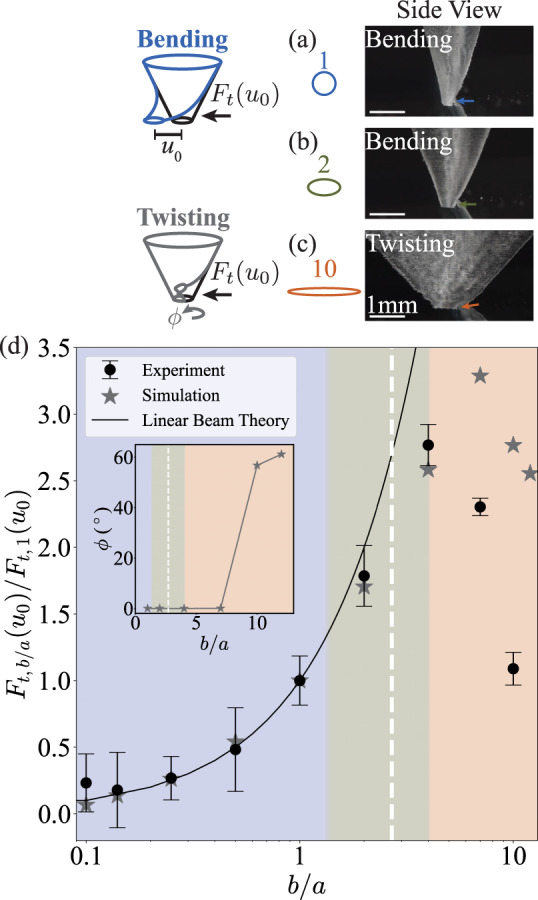
Moderately elliptical prickles are relatively good at resisting shear. The bending characteristics of tapering PDMS beams (aspect ratio b/a) were explored by application of a tangential force $F_{t,b/a}(u_{0})$ at the tip. The force was increased until a displacement $u_{0}=0.4\;$mm along the x-axis was reached. (This value of $u_{0}$ corresponds to the tip radius of the circular stinger; table S2 in the supplementary material.) (a–c) Circular and moderately eccentric stingers (e.g. b/a=1 and 2) bend along their entire length. By contrast, the tips of slender stingers (e.g. b/a=10) twist at the tip. (d) This transition is also apparent when plotting the force $F_{t}$ as a function of aspect ratio b/a. Experimental data (black dots) and numerical simulations (grey stars) highlight that the force increases monotonically with the aspect ratio b/a until b/a≈4. This is consistent with beam theory ($F_{t}∼b/a$, solid line). However, beyond this point, the tip begins to twist. Since the displacement $u_{0}$ remains constant, the reaction force decreases. Notably, the twisted blade edge no longer aligns with the cutting axis, potentially diminishing its effectiveness. This picture is relatively insensitive to the choice of displacement $u_{0}$ when $u_{0}$ is between 50% and 250% of the circular tip diameter (figure S5 in the supplementary material). The inset in (d) shows the torsion angle ϕ for aspect ratios b/a≥1 obtained from the simulations. For an imposed shift $u_{0}$ along the x-axis, the orthogonal tip displacement v (along the y-direction) was recorded corresponding to the torsion angle $\phi =\arctan\left(v/u_{0}\right)$. Numerical data suggest that the torsion angle ϕ increases with the aspect ratio b/a. This phenomenon could, in part, explain the deviation of the normalized force from the linear beam theory. The dashed vertical line indicates the mean biological aspect ratio b/a=2.7, and the background is shaded using the colour codes introduced in figure 1b. Note that in (d), the force data are normalized to a circular prickle (b/a=1); see also figure S4 in the supplementary material.

Creating lacerations requires a structure capable of resisting shear. We therefore first consider bending and focus on the tangential force $F_{t}(u,b/a)$ required to displace a stinger of aspect ratio b/a a distance $u_{0}$ corresponding to the tip radius (figure 3; table S2 in the supplementary material). Here, data indicate a linear dependence on the eccentricity, $F_{t}(u_{0},a/b)/F_{t}(u_{0},1)\approx b/a$, for b/a≤4. This is consistent with Euler buckling, since the inertial moment of an elliptical beam is a factor b/a times the moment of a circular beam of equal area [35] (see supplementary material, §S10, for a detailed description of the dependence of linear beam theory on the aspect ratio). Computer simulations support this conclusion. We note that no fitting parameters were used to match experiments, simulations and theory in figure 3. However, all force measurements were normalized by the response of the circular control prickle (b/a=1), which allows direct comparison with the parameter-free scaling prediction from beam theory. As detailed in the supplementary material, our finite-element model is not able to quantitatively predict the force experienced by the prickle, while it is capable of representing the main physics at play via a qualitative match of the dimensionless forces as shown in figure 3.

An interpretation of the data in figure 3 is that deviating from the circular cross-section allows prickles to provide a greater reaction force under the same displacement. Notably, however, data deviate from linearity when b/a>4. Here, twisting of material near the tip dramatically decreases the tangential force. This occurs as the tip appears to buckle in the y-plane, that is perpendicular to the cutting direction. The peak tangential force was observed for b/a=4, which is nearly three times stronger than the circular (b/a=1) tapering beam. We note that the biomimetic force optimum is less than two standard deviations from the biological mean. Numerical simulations provide further insight into this transition. By extracting the torsion angle of the prickle tip from the 3D bending simulations, we observe a clear shift from bending-dominated deformation to torsion-dominated deformation as the aspect ratio increases. In the simulations, this transition occurs around b/a∼6, slightly above the experimental value b/a∼4. The discrepancy is likely attributable to small geometric imperfections or alignment variability in the physical specimens, which are known to reduce the critical threshold for instability in slender structures. Despite this modest shift, both experiments and simulations exhibit the same qualitative behaviour: an initial increase in tangential stiffness with aspect ratio, followed by a reduction once torsional deformation becomes dominant.

**Figure 4.**
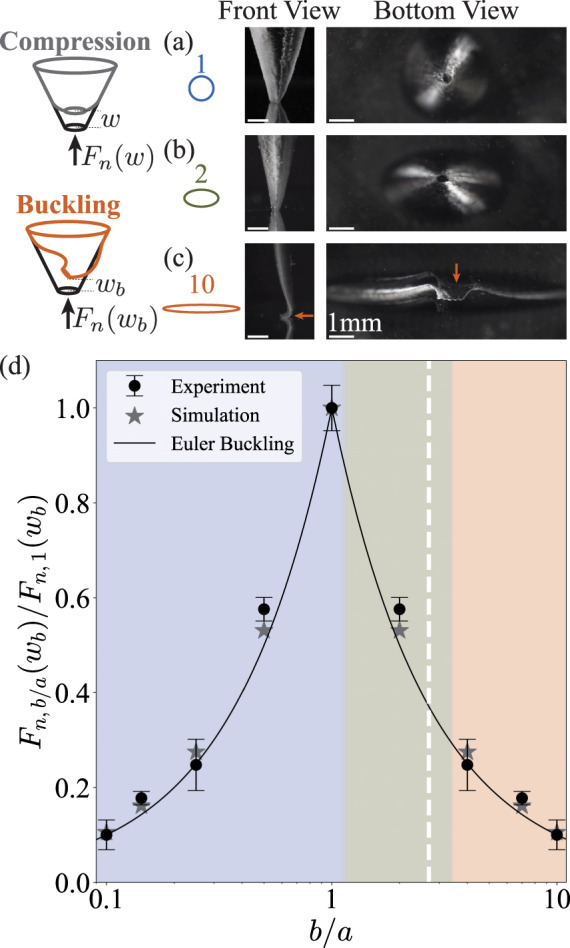
Elliptical prickles are relatively poor at resisting buckling. The buckling characteristics of tapering PDMS beams (aspect ratio b/a) were explored by imposing a vertical displacement w along the z-axis. The displacement was increased until buckling ($w=w_{b}$), and the force $F_{n,b/a}(w_{b})$ was recorded. (a–c) The dynamics for a fixed displacement w=0.4 mm, corresponding to one tip diameter of the b/a=1 stinger. Both the circular (b/a=1) and the moderate (b/a=2) oval shapes retain their form. However, the slender morphology (b/a=10) buckles along the weakest axis. (d) This picture is apparent when plotting the buckling force $F_{n,b/a}(w_{b})$ as a function of the aspect ratio b/a. Experimental data (black dots) and numerical simulations (grey stars) highlight that the force increases linearly with the aspect ratio b/a. This is consistent with beam theory (solid line). The dashed vertical line indicates the mean biological aspect ratio b/a=2.7, and the background is shaded using the colour codes introduced in figure 1b. Note that in (d), data and simulation results are symmetric around b/a=1. Force data are normalized to the circular prickle (b/a=1); see also figure S5 in the supplementary material.

Cutting also involves normal forces, and these may cause the stinger (and tissue) to deform. However, creating lacerations requires a structure capable of resisting buckling. This abrupt change in form occurs when a column is subjected to compressive (normal) stress beyond its ability to support the load without lateral movement. Data demonstrate (figure 4) that the critical buckling force $F_{c}(b/a)$ scales inversely with the aspect ratio, that is, $F_{c}(b/a)/F_{c}(1)∼a/b$, consistent with Euler buckling theory for the reasons outlined above [36]. Computer simulations again support this conclusion in terms of the dimensionless force ratios, while a quantitative agreement in terms of the absolute force experienced by the prickle is still lacking.

Extrapolating these findings to the mechanical properties of biological samples should be done with caution since many factors can influence the results (see Discussion). Nevertheless, we can state that the oval morphology of rose prickles (mean aspect ratio b/a=2.7) permits them, in principle, to increase shear forces by 170% when compared to a circular stinger. This comes at the cost of buckling resistance, with the threshold load reduced by 63%.

## 4. Discussion and conclusion

By contrast to most natural stingers, which are circular, the majority of rose prickles have an oval cross-section with an aspect ratio b/a=2.7±0.7 (figure 1). Our analysis provides insights into factors that could, at least partially, explain this unique design. We have explored potential factors that may decrease the fitness of stingers outside the empirically observed range, that is prickles that are either circular (b/a=1) or strongly eccentric (very wide, b/a≫1, or very narrow, b/a≪1). Circular prickles tend to bend in the direction of travel and are inefficient in creating lacerations and providing grip. By contrast, moderate aspect ratio prickles (b/a=2--4) appear to have the mechanical stability to both cut and provide adhesion. In narrow stingers (b/a>4), however, the blade becomes so weak that it buckles. (This also holds when attempting to cut along the weak axis, i.e. when b/a<1.)

The biomimetic system employed here does not reproduce the full physiological complexity of interactions between rose prickles and animal skin. Instead, it is designed to capture the dominant mechanical processes governing deformation, bending and buckling of the prickle. In such physical model systems, similarity of the governing mechanical mechanisms is generally more important than exact matching of material properties. In particular, the interaction is controlled by the elastic modulus of the prickle material, the stiffness of the substrate and the geometry of the cutting structure. In our experiments, PDMS has a Young’s modulus of the order of megapascals, whereas gelatin has a modulus in the tens of kilopascals range, yielding a stiffness contrast of roughly two orders of magnitude. This contrast is not broadly inconsistent with the mechanical mismatch between plant structural tissues and soft biological tissues. Maintaining these relationships allows the qualitative mechanical regimes (bending, cutting, buckling) to be reproduced in a controlled laboratory system.

The observed morphology could thus be a trait that protects against herbivory, and, additionally, it allows the plant to climb, both factors with the potential to enhance the organism’s survival chances. This interpretation of the biological data warrants some caution. While the observed aspect ratio distribution is consistent with the mechanically favourable regime identified here, it does not constitute direct evidence of adaptive selection. Alternative explanations—including sampling bias, phylogenetic constraints or developmental limitations—could also contribute to the observed pattern [30]. A rigorous test of adaptive hypotheses would require larger datasets, phylogenetic comparative methods and explicit null models for trait evolution. The present work instead identifies a region of morphospace that is mechanically unfavourable for cutting and grip, thereby providing a physically grounded explanation for why certain geometries may be disfavoured. In this sense, our results are best interpreted as delineating feasible versus unfavourable designs, rather than demonstrating optimality.

Several other important caveats warrant discussion. For instance, the precise size, shape and material properties of both artificial prickles and the biomimetic skin are not representative of all rose varieties and their environments. These factors could play a significant role in setting the precise thresholds for transitioning between the physical regimes discussed here. Additionally, the material properties of prickles do not follow the isotropic elastic model used here. Instead, they are known to have hierarchical characteristics with, for example, strong outer layers and soft inner structures [15,37]. This structure could impact both the stress–strain behaviour and may modify the absolute force thresholds for cutting, bending and buckling. More complex material architectures could also shift or broaden the optimal range of aspect ratios, a question worth exploring in future studies using biologically realistic composite materials. Similarly, prickles are often curved, an effect not considered here, in part because there is a minimal impact of structural curvature in biological puncture mechanics [38]. Nevertheless, such hook-like geometries can generate directional friction and anchoring effects during sliding contact, as observed in several biological attachment systems. For example, curved hooks in climbing plants [16] and animal quills [39] can produce asymmetric resistance to motion depending on the direction of travel. In roses, the downward orientation of prickles has often been associated with climbing or anchoring onto neighbouring vegetation or rough surfaces [16]. In the present study, we focused on the influence of cross-sectional eccentricity on bending and buckling stability while keeping other geometric parameters constant. This allowed us to isolate the role of elliptical cross-sections in cutting mechanics. Because the curvature of natural prickles typically occurs over length scales comparable to the prickle length rather than the local tip geometry that governs cutting initiation, we expect it to have a secondary effect on the onset of laceration. Nevertheless, curvature may influence the directionality of friction and tearing during sliding contact, and incorporating such geometrical features would be an interesting extension of the present framework.

Additionally, details of interactions between a specific plant and biotic or abiotic factors might also differ significantly from the conditions outlined here. Our focus was on tangential motion relative to soft surfaces that might provide grip or create lacerations. This differs from processes that involve normal contact, for example tissue penetration. Plant–animal interactions can also involve more complex loading scenarios than the tangential configuration studied here. For example, prickles may experience oblique contact with teeth, sliding interactions with fur or compressive loading when an animal presses against the stem, and the resulting deformation and fracture depend strongly on the material properties and loading geometry of the contacted substrate [40,41]. In such situations, the local forces acting on a prickle can generally be decomposed into tangential and normal components. The mechanical trade-offs identified in this study are, therefore, expected to remain relevant, since they are governed primarily by the anisotropic bending stiffness of the elliptical cross-section. Circular prickles remain susceptible to bending under shear-dominated loading, whereas strongly eccentric prickles become increasingly vulnerable to buckling along their weakest axis under compressive forces. The intermediate aspect ratios observed in roses may, therefore, provide a compromise that maintains structural stability across a range of ecological interactions beyond the specific tangential configuration explored in our experiments.

In summary, the suggestion that physical constraints discourage circular and highly eccentric prickles aligns with available data. We hypothesize that these design principles could be applied in technical fields that require grip, such as gloves and tyres.

## Supplementary Material



## Footnote

^1^
Rose prickles can deliver bacteria and fungi into the skin and cause infection [17]. To protect oneself, we recommend the wearing of personal protective equipment, like gloves.

## Data accessibility

Data are available at Zenodo [42].
