# Supplementary material for: Mechanical limits shape the eccentric form of rose prickles
Source: J R Soc Interface. 2026 Aug 12;23(241):20251300. doi: 10.1098/rsif.2025.1300 (PMC13479369; doi:10.1098/rsif.2025.1300)
Supplement: Supplementary file 1 [file rsif.2025.1300.zip › rsif20251300/rsif20251300_si_001.pdf]

ELECTRONIC SUPPLEMENTARY MATERIAL

S1. Biological Prickles

Data from 19 varieties of roses were collected at *Landbohøjskolen Have* in Copenhagen, Denmark (Fig. S1). Four to six prickles were collected from each variety. The base diameters were measured and aspect ratios calculated. The mean aspect ratio b/a and the standard deviation are provided in Table S1 and Fig. S2.

S2. Biomimetic prickles

Prickles of different cross-sections were designed with a constant volume, V, total length, L, and contact area, A_t (see Fig. 1a, Fig. S3, and Tab. S2). A mold of the negative prickle was 3D printed (Prusa MK4, Prusa Research a.s, Czech Republic; nozzle 0.25mm) in PLA. Polydimethylsiloxane (PDMS, Sylgard 184 Silicone Elastomer, Dow, USA) was mixed with the standard 10:1 ratio of base to curing agent. The polymer was put under vacuum for 30min, before filling the molds. After another 5-10 min under vacuum, the PDMS was cured for at least 24h at 40°C. The prickles with aspect ratio $b/a < 1$ were obtained from the corresponding prickle of $b/a > 1$ rotated by 90°.

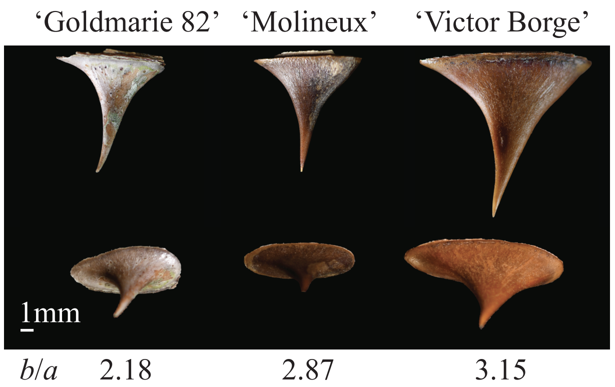

FIG. S1. Photographs illustrating rose prickle shape diversity.

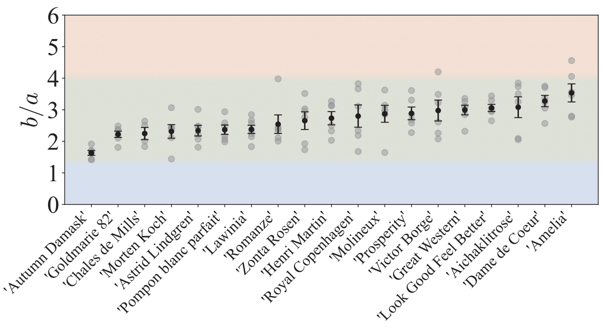

FIG. S2. Rose prickle morphology. Varieties are sorted by the aspect ratio b/a, see Tab. S1 .

TABLE S1. Anatomical data for rose prickles. The number (No.), Name of rose variety and aspect ratio (b/a) with standard deviation from a minimum of four measurements are given, as shown in Fig. 1 and Fig. S2.

No.	Variety	b/a
1	Autumn Damask	1.63 ± 0.08
2	Goldmarie 82	2.22 ± 0.10
3	Chales de Mills	2.25 ± 0.19
4	Morten Koch	2.31 ± 0.22
5	Astrid Lindgren	2.34 ± 0.17
6	Pompon blanc parfait	2.37 ± 0.15
7	Lawinia	2.38 ± 0.13
8	Romanze	2.54 ± 0.30
9	Zonta Rosen	2.66 ± 0.28
10	Henri Martin	2.73 ± 0.21
11	Royal Copenhagen	2.80 ± 0.35
12	Molineux	2.87 ± 0.27
13	Prosperity	2.89 ± 0.20
14	Victor Borge	2.98 ± 0.33
15	Great Western	2.99 ± 0.15
16	Look Good Feel Better	3.06 ± 0.11
17	Aichaklitrose	3.08 ± 0.33
18	Dame de Coeur	3.28 ± 0.18
19	Amelia	3.54 ± 0.29

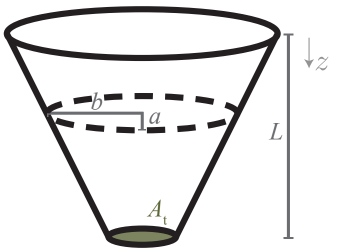

FIG. S3. Schematic illustration of the biomimetic prickle. See parameter definitions in Tab. S2.

TABLE S2. Geometric values of all model prickles. Schematic illustration of parameters is shown in Fig. S3.

	Prickle 1	Prickle 2	Prickle 4	Prickle 7	Prickle 10
b/a	1	2	4	7	10
a_0 (mm)	3.2	2.3	1.6	1.2	1.0
b_0 (mm)	3.2	4.6	6.5	8.6	10.3
a_L (mm)	0.4	0.3	0.2	0.2	0.1
b_L (mm)	0.4	0.6	0.8	1.1	1.3
A_t (mm^2)	0.6	0.6	0.6	0.6	0.6
L (mm)	10.0	10.0	10.0	10.0	10.0
V (mm^3)	126.5	126.5	126.5	126.5	126.5

S3. Gelatin preparation

Gelatin preparation was adapted from [37]. Gelatin (10% wt Bloom 220; Condi ApS, Denmark) was mixed and kept at 80°C for 1 h before being poured into a petri dish and allowed to set at room temperature for 1h. Afterward, the gelatin was kept at 20°C for 24-28h before use.

S4. Elastic modulus

Elastic moduli of gelatin (10%wt Bloom 220) and PDMS (10:1) were measured by obtaining the extension of a rectangular slap of the material under additional weight. The resulting stress-strain curves were fitted using a linear regression, the slope of which is equal to the Young's modulus. The Young's modulus for gelatin was measured to be 18.8 ± 0.5 kPa. The one for PDMS was measured to be 1.7 ± 0.3 MPa. Standard deviations are calculated from repeating the experiment three times. In the literature, the Young's modulus of gelatin ranges from 10-100 kPa [38, 39] and the modulus of PDMS lies between 1.32 and 2.05 MPa [40]. Our measured values are therefore within the expected ranges.

S5. Cutting

A micro manipulator (original design from [20]) was used to position the prickle directly above a gelatin sample. The prickle was then moved diagonally (∼27° angle; 2.75 mm) into the gelatin, followed by a horizontal movement of 5 mm. Once the cutting process was completed, the micro manipulator was used to move the prickle vertically up and away from the substrate.

Videos were taken with a Nikon camera (D5300, Nikon, Japan) with a Nikon lens (Series E, 50 mm; 1:1.8) that was used inverted. The camera was positioned in the front, on the side, or with a mirror (placed at 45 ° beneath the gelatin sample) to capture the bottom view (see Fig. 1d). A camera (D7500, Nikon, Japan; with lens AF-S Micro NIKKOR 105mm1:2.8G ED) was used to take a picture of the gelatin after cutting. A binary score was given to indicate a cut (defined as a breach of the surface; pure abrasions on the surface did not count as a cut). The length of the cut (ℓ_c) was measured. The length was set to zero if no cut was observed. All experiments were repeated at least six times and standard deviations were determined from these repetitions.

The specific number of repetitions (n) per prickle with cross-sectional aspect ratio b/a, along with the number of times a prickle initiated a cut (n_C) are given in Table S3. Binomial tests were performed and showed significant differences of our experimental results (see Table S3).

TABLE S3. Results of binomial test for the probability of cutting in shown in Fig. 2. For each aspect ratio b/a the total number of replicas, n as well as the number of times the prickle initiated a cut n_C are given. Additionally, binomial tests were performed for three different null hypotheses. H1: The prickle cuts 100 % of the time. H2: The prickle cuts 50 % of the time. H3: the prickle cuts 0 % of the time. The results of the binomial test are given as not significant (ns) for a p-value ≥ 0.05. For p-values lower than that, significance levels are marked as (*) for a p-value < 0.05, (**) for a p-value < 0.01 and (***) for a p-value < 0.001.

b/a	n	n_C	H1	H2	H3
0.1	6	0	***	*	ns
0.14	6	0	***	*	ns
0.25	6	0	***	*	ns
0.5	6	0	***	*	ns
1	9	4	***	ns	***
2	6	6	ns	*	***
4	9	8	***	*	***
7	6	4	***	ns	***
10	8	2	***	ns	***

S6. Buckling experiments

A micro manipulator (original design from [20]) was used to vertically move the prickles onto a hard substrate (glass slide) while measuring the force exerted in the vertical direction using a load cell (mini load cell 100g straight bar, 1568-14727-ND, SparkFun Electronics, USA). Videos were taken from the front and bottom views in the same way as for the cutting experiments. All measurements were repeated three times. The buckling force was extracted from the force-displacement curves, where the slope changes (see Fig. S4). If the slope change could not be clearly identified, a video inspection was performed and used to find the displacement at which the buckling occurred.

S7. Buckling simulations

We have run 2D solid mechanics simulations in COMSOL Multiphysics (v. 6.2), within the Solid Mechanics interface. We have modeled the prickle as a linear elastic material with a Poisson ratio $\nu = 0.45$ (as close as possible to incompressibility) and a Young's modulus $E = 1.7$ MPa. We have assumed the prickle to be in a state of plane strain and to be on a perfectly rigid substrate. The contact has been simulated via a penalty method, adding a Coulomb friction with a friction coefficient of 0.3, a cohesion sliding resistance of 20 Pa, and an infinite maximum tangential fraction. A vertical displacement has been imposed on the top surface. For each aspect ratio, we have lowered the prickle until contact was established, and then even further to observe the buckling behavior. As the simulations were displacement-controlled, the vertical force was computed as the reaction force at

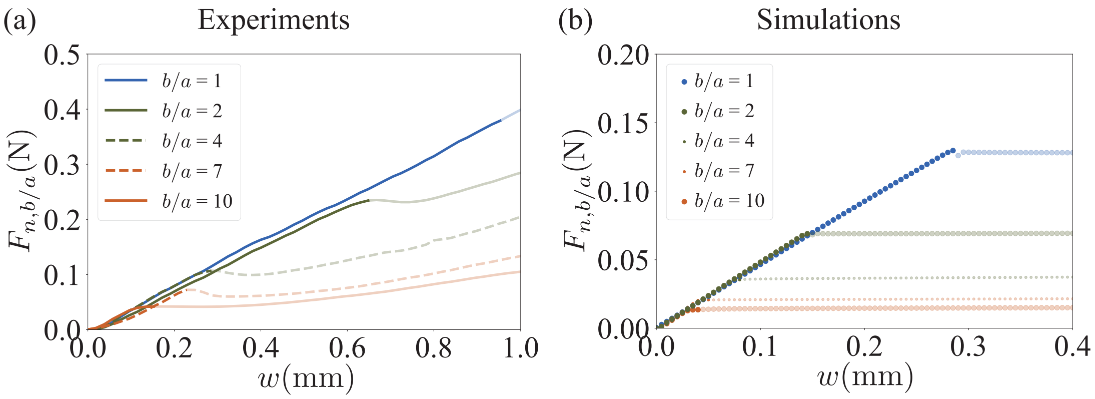

FIG. S4. Force-displacement curves for buckling of (a) model PDMS prickles and (b) simulations. The data is separated in before (color data) and after (transparent data) buckling.

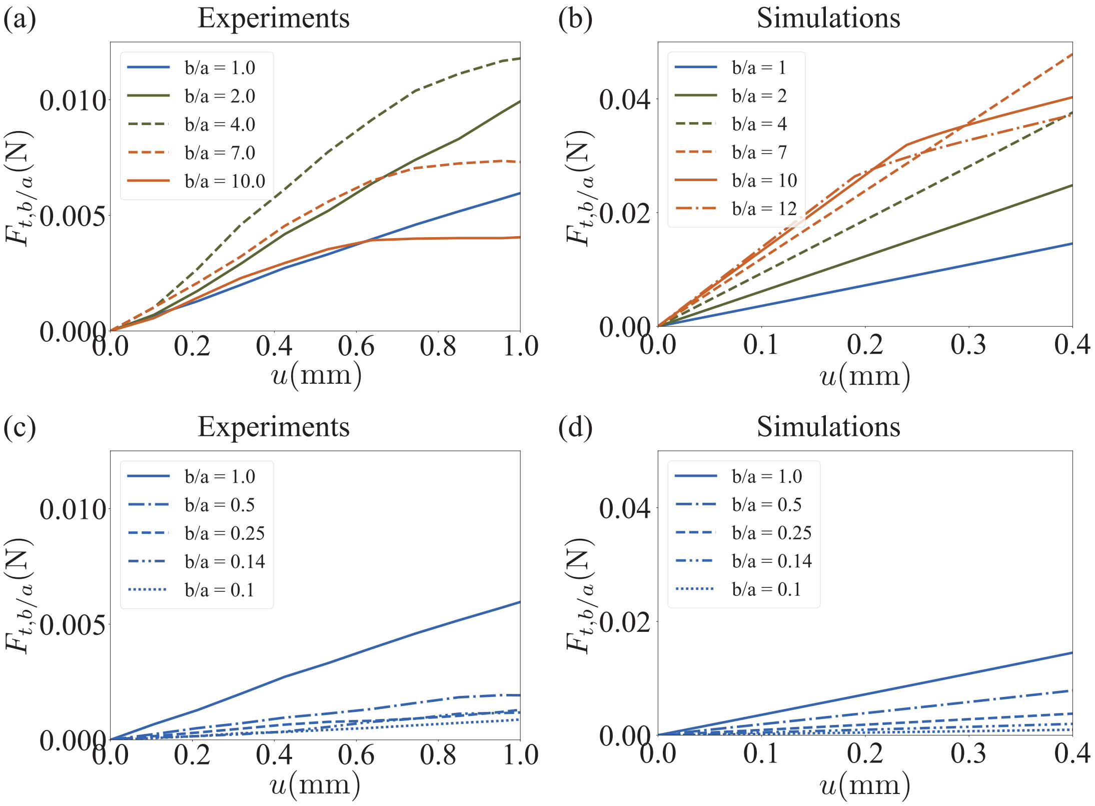

FIG. S5. Force-displacement curves for bending of (a,c) model PDMS prickles and (b,d) simulations.

the top surface of the prickle. Moreover, since the geometry is 2D, we obtained the force by multiplying the force per unit width by the average width of the cross-section, that is $\pi/4b_1$. Quadratic serendipity shape functions were used and a convergence analysis led to a mesh of around 10^5 DOFs, depending on the specific geometry. Fig. S4 shows the comparison between experiments and simulations for the buckling tests, showing a lack of quantitative agreement. Indeed, our 2D simulations cannot fully represent the 3D experiments, as for example the cross-section of the prickle changes as we move along the height of the prickle. Nevertheless, as Fig. 3 in the main text shows, the FE model successfully describes the main physics at play.

S8. Bending

A micro manipulator (original design from [20]) was used to move the tip of the prickles horizontally towards a hard substrate while measuring the tangential force using a load cell (mini load cell 100g straight bar, 1568-14727-

ND, SparkFun Electronics, USA). Videos were taken from the side view in the same way as for the cutting experiments. All experiments were repeated three times. Bending forces were extracted at a displacement of $u_0 = 0.4$ mm from the force-displacement curves (see Fig. S5).

S9. Bending simulations

Bending simulations were carried out within the Solid Mechanics interface and with the same material parameters used for the buckling simulations, but within a 3D framework and without any contact mechanics. Simulations were displacement-controlled, with a displacement imposed either in the y or x direction at the bottom surface of the prickle via a prescribed displacement node. The corresponding force was calculated as the reaction force at the top base of the prickle. Quadratic serendipity shape functions were used and a convergence analysis led to a mesh of around 10^5, depending on the specific geometry.

For prickles of aspect ratio $b/a \geq 1$ a torsion angle was determined from the simulations. The displacement of the prickle tip vertical to the applied force (v) was measured at a displacement $u_0 = 0.4$ mm allowing for the torsion angle to be calculated as $\arctan(v/u_0)$ (see Fig. 3).

S10. Linear Beam Theory and Euler Buckling

We used linear beam theory and Euler buckling to obtain a scaling law between the normalized force ($F_{b/a}/F_1$) in bending and buckling, respectively, and the aspect ratio b/a. For bending, the tangential load (F_t) on a slender cylinder with one fixed end and small deflections ($u \ll L$) can be described using the Euler-Bernoulli equation [29]

$$F_t = EI\frac{d^4u}{dz^4}. \tag{S2}$$

Here, E is the Young's modulus of the beam, I is the moment of inertia, and u the displacement in the direction of the tangential force, corresponding to our x-axis, at the prickle tip ($z = L$). The determining factor in the normalized force is the moment of inertia which depends on the cross-section. For a circular beam, I_1 is given as [41]

$$I_1 = \frac{\pi}{4}r^4, \tag{S3}$$

where r is the radius of the circle. For an elliptical beam of aspect ratio b/a the moment of inertia $I_{b/a}$ is given by

$$I_{b/a,x} = \frac{\pi}{4}a^4\frac{b}{a} \tag{S4}$$

in the x-plane or

$$I_{b/a,y} = \frac{\pi}{4}a^4\left(\frac{b}{a}\right)^3 \tag{S5}$$

in the y-plane [42]. Considering that the area of the circle and the area of the ellipse are equal, the radius of the circle can be expressed as $r = a(b/a)^{1/2}$. In our set-up, we apply the tangential force along the x-axis, which means the deflection of the beam occurs in the y-plane. The normalized force at a specific deflection u_0 ($F_{t,b/a}(u_0)/F_{t,1}(u_0)$) is then given as

$$\frac{F_{t,b/a}(u_0)}{F_{t,1}(u_0)} = \frac{b}{a} \tag{S6}$$

for all a, b. This expression is plotted in Fig. 3 to be used as a scaling model for bending at different aspect ratios. We note that linear beam theory is successful in describing the bending behavior of the prickles, because the maximum force is measured for $u = 0.4$ mm, which corresponds to small strains since $u/L = 0.04$.

When considering buckling of a slender beam, the critical normal force (F_n) that has to be applied can be described by Euler's critical load [30]

$$F_n = \frac{\pi^2 EI}{L^2}. \tag{S7}$$

As before, E is the Young's modulus and I is the moment of inertia. L is the length of the beam. Both the length and the Young's modulus are kept constant in between circular and elliptical beams. Buckling occurs in the plane of lowest moment of inertia, meaning when $b > a$ the beam will buckle in the x-plane and when $b < a$ it will buckle in the y-plane. Therefore, the normalized buckling force at the corresponding displacement $w = w_b$ ($F_{n,b/a}(w_b)/F_{n,1}(w_b)$) is given as

$$\frac{F_{n,b/a}(w_b)}{F_{n,1}(w_b)} = \frac{b}{a}, \tag{S8}$$

for $b > a$ and as

$$\frac{F_{n,b/a}(w_b)}{F_{n,1}(w_b)} = \frac{a}{b} \tag{S9}$$

for $b < a$. These expressions are mirrored around an aspect ratio of 1 and are used as the scaling model in Fig. 4 describing buckling at different aspect ratios.
